# Bio-Based PLA/PBS/PBAT Ternary Blends with Added Nanohydroxyapatite: A Thermal, Physical, and Mechanical Study

**DOI:** 10.3390/polym15234585

**Published:** 2023-11-30

**Authors:** Pei-Hua Chen, Chin-Wen Chen, Hsu-I Mao, Chi-An Dai, Chie-Shaan Su, Jung-Chin Tsai, Feng-Huei Lin

**Affiliations:** 1 Department of Biomedical Engineering, National Taiwan University, Taipei 106319, Taiwan; d10528004@ntu.edu.tw; 2 Department of Orthopedics, Shuang Ho Hospital, Taipei Medical University, New Taipei City 235041, Taiwan; 3 Department of Molecular Science and Engineering, Institute of Organic and Polymeric Materials, Research and Development Center of Smart Textile Technology, National Taipei University of Technology, Taipei 106344, Taiwan; eeric0212@hotmail.com; 4 Department of Chemical Engineering, Institute of Polymer Science and Engineering, National Taiwan University, Taipei 106319, Taiwan; polymer@ntu.edu.tw; 5 Department of Chemical Engineering and Biotechnology, National Taipei University of Technology, Taipei 106344, Taiwan; cssu@ntut.edu.tw; 6 Department of Chemical Engineering, Ming Chi University of Technology, New Taipei City 243303, Taiwan; jctsai@mail.mcut.edu.tw

**Keywords:** PLA, PBS, PBAT, nHA, polymer blends, mechanical strength, physical properties

## Abstract

The physical and mechanical properties of novel bio-based polymer blends of polylactic acid (PLA), poly(butylene succinate) (PBS), and poly (butylene adipate-co-terephthalate) (PBAT) with various added amounts of nanohydroxyapatite (nHA) were investigated in this study. The formulations of PLA/PBS/PBAT/nHA blends were divided into two series, A and B, containing 70 or 80 wt% PLA, respectively. Samples of four specimens per series were prepared using a twin-screw extruder, and different amounts of nHA were added to meet the regeneration needs of bone graft materials. FTIR and XRD analyses were employed to identify the presence of each polymer and nHA in the various blends. The crystallization behavior of these blends was examined using DSC. Tensile and impact strength tests were performed on all samples to screen feasible formulations of polymer blends for bone graft material applications. Surface morphology analyses were conducted using SEM, and the dispersion of nHA particles in the blends was further tested using TEM. The added nHA also served as a nucleating agent aimed at improving the crystallinity and mechanical properties of the blends. Through the above analyses, the physical and mechanical properties of the polymer blends are reported and the most promising bone graft material formulations are suggested. All blends were tested for thermal degradation analysis using TGA and thermal stability was confirmed. The water absorption experiments carried out in this study showed that the addition of nHA could improve the hydrophilicity of the blends.

## 1. Introduction

In recent years, the research and development of biomaterials have developed rapidly to meet the needs of tissue engineering to improve human life. The interface between synthetic biology and biomaterial design creates promising challenges in health, biotechnology, and sustainability [1]. Proper design of biomaterials must cover both their mechanical properties and biological activity. Future biomaterials can maintain organ transport and self-healing functions, biocompatibility, biodegradability, bioactivity, and tissue regeneration activity [1,2]. Biomaterials are generally classified as natural, metal and alloy-based, ceramic-based, polymer-based, or composite materials for tissue engineering scaffolds, each of which has a variety of applications [3,4]. It has been reported that surgeries to treat bone defects in orthopedics has a growing rate [5], and the development of biomaterials for orthopedic implants has received much attention [6,7,8,9,10]. The design and fabrication techniques for bone tissue engineering have been reported in the review literature, addressing engineering processes and functional properties [11,12,13,14]. Although most bone implants are metal-based, polymers and their composites are projected to become more suitable in the future [15]. In recent reviews, next-generation bone tissue bio-based materials, especially biodegradable polymers and nanotechnology, have received much attention [16,17,18].

Polylactic acid (PLA) is the most commonly used semi-crystalline bio-based polymer approved by the U.S. Food and Drug Administration (FDA) for its desired biocompatibility and biodegradability [19]. In order to improve some of its limitations in terms of mechanical properties, toughness, hydrophilicity, and cell affinity, composites of PLA with other polymers or inorganic additives have been studied in the literature. For example, hydroxyapatite (HA) has been added to PLA to study the in vitro osteogenic differentiation of human mesenchymal stem cells [20]. According to previous reports [21,22], the current improvement of tough PLA was achieved through polymer blending, copolymerization, plasticization, or composite modifications. Blending PLA with other elastomeric polymers is relatively easy and often considered to be the most effective method. PLA with biodegradable polymers such as polycaprolactone (PCL), poly(butylene succinate) (PBS), poly(butylene succinate-co-adipate) (PBSA), and poly(butylene adipate-co-terephthalate) (PBAT) have been discussed. Recent literature has reported the mechanical and physical properties of blends of PBS and PBAT with various compositions, including behavior after supercritical CO_2_ foaming [23,24]. The mechanical and physical properties of PLA/PBS or PLA/PBAT blends with proper compositions have been investigated in previous reports, demonstrating their morphology, mechanical properties, and potential applications as scaffolds in tissue engineering [25,26,27,28,29,30,31]. Biomaterials for tissue engineering preferably contained bioactive components such as chitosan (a natural polysaccharide) or HA (the main component of human bone tissue) in structured scaffolds to facilitate the regenerative process. The fabrications of PLA-based scaffolds with other biodegradable polymers and bioactive materials have been shown in the literature [32,33,34,35,36,37,38]. It has been reported that loaded nano-HA (nHA) particles could promote cell proliferation and mimic the structure of the bone extracellular matrix [20,35,36,37,38,39].

To the best of our knowledge, most literature reports have presented the properties and applications of biodegradable binary polymer blends (for example, PLA/PBS or PLA/PBAT) [28,29,31] or a single polymer with nHA particles added [36,40,41]. Although some studies on ternary systems have been carried out [38,40,41,42], more experimental measurements are still needed. The aim of this study was to explore the properties of novel polymer blends containing three biodegradable polymers (PLA, PBS and PBAT) with inorganic nHA added. The main component of the blends was PLA, which is suitable for bone graft applications due to its good mechanical strength. The elongation and toughness limits of PLA can be improved by blending with ductile PBS and PBAT. Biodegradable PBS also showed good cell viability [38]. PBAT has a strong but underexploited potential for bone growth in vivo, and the studies on including nHA in PBAT are still limited [35,36]. Therefore, this study proposes a novel bone graft biomaterial, a PLA/PBS/PBAT/nHA composite, produced using a twin-screw extruder. No organic solvent was used in this study to avoid any safety concerns as implant biomaterials. The mechanical and physical properties of various formulations of this blend were measured and promising blends for further bone graft applications are illustrated.

## 2. Materials and Methods

### 2.1. Materials

Polylactic acid (PLA, Ingeo 4032D) was purchased from NaturalWorks LLC, Minnetonka, MN, USA. It is semi-crystalline, with an average D-lactide content of 1.4 wt%. Poly(butylene succinate) (Bio PBS, FZ 91), a semi-crystalline polyester, was purchased from PTT MCC Biochem Co. Ltd., Bangkok, Thailand. Poly(butylene adipate-co-terephthalate) (PBAT, ecoflex F blend C1200) was purchased from BASF SE, Ludwigshafen, Deutschland. The physical and mechanical properties of these polymers are listed in Table 1. Nano-hydroxyapatite (nHA, CAS registry number 12167-74-7) was purchased from Sigma-Aldrich, UNI-ONWARD Corp., Taiwan, with purity greater than 97 wt%, mean particle size of 72–80 nm, molecular weight of 502.3, and melting temperature of about 1100 °C.

### 2.2. Preparation of Composite Blends

PLA, PBS, and PBAT polymers were first dried in a vacuum oven at 80 °C for 6 h before compounding. These blends were prepared using a twin-screw extruder (Process 11, Thermo Fisher Scientific, Waltham, MA, USA). The extruder was equipped with a volumetric feeder and a strand pelletizer for blending the polymers. The diameter of the screw extruder is 11 mm, with an L/D ratio of 40. Polymers were fed into the hopper of the extruder for the compounding process. The extruder has six zones, from feed to die, where the extrusion temperatures were controlled independently. The feed rate of the polymer pellets was 1 kg h^−1^, and the screw speed was 50 rpm. The temperature settings for the feed and mixing zones were adjusted to be 200–210–220–225–225–220 °C. After blending, a water bath was used to cool the extruded products. These products were then granulated and dried for a sufficient time before mechanical property testing. Plate specimens with a thickness of 1.2 ± 0.2 mm were made by compression molding at 200 °C for 5 min, followed by cooling to room temperature. The specimens were packed in plastic bags and stored in cool surroundings before tests. The extruded products for polymers with added nHA were prepared with the same procedure. Samples with various formulations were also used for thermal, chemical composition, morphology, and water absorption analyses.

Table 2 lists the formulations of PLA-based polymer blends in this study. The specimen code PLA60 represented a polymer blend containing 60 wt% PLA and two other polymers (25 wt% PBS and 15 wt% PBAT), without the addition of nHA. Sample series A and B listed in Table 2 included specimen codes with various amounts of nHA added. For example, specimen A0 listed in Table 2 represented a blend containing 70 wt% PLA, 20 wt% PBS, 10 wt% PBAT, and no added nHA. Specimens A3, A5, and A7 in sample series A had the same PLA/PBS/PBAT composition as specimen A0, but with the addition of 3, 5, and 7 wt% nHA, respectively. Sample series B contained 80 wt% PLA, 15 wt% PBS, 5 wt% PBAT, with 0, 3, 5, and 7 wt% nHA added to specimens B0, B3, B5, and B7, respectively. It has been pointed out that nanocomposites comprising nHA favored the biocompatibility of bone formation [10,36,37,38,39,43,44]. The average particle size of nHA used in this study was within the range of nHA particle sizes reported in the literature [44].

### 2.3. Analysis and Testing

#### 2.3.1. Fourier-Transform Infrared Spectroscopy (FTIR)

Fourier-transform infrared spectroscopy (FTIR, Thermo Fisher Scientific Summit LITE Spectrometer, Madison, WI, USA) using the ATR technique was employed for analyzing the chemical structures of the polymer blends. The wavenumber in the tests ranged from 4000 to 500 cm^−1^. The resolution was 4 cm^−1^, and the number of scans was 32.

#### 2.3.2. X-ray Diffraction Analysis

The intensity of crystallinity of the polymer blends was analyzed using an X-ray diffractometer (XRD, Rigaku, Tokyo, Japan) with Cu-K_α_ radiation (λ = 1.54 Å) operated at 40 kV and 15 mA. The XRD data were collected in the 2θ angle range from 5 to 40°, with a scanning rate at 10° min^−1^.

#### 2.3.3. Differential Scanning Calorimeter Measurements

The thermal and crystallization behavior of various polymer blends was analyzed using differential scanning calorimetry (DSC, Hitachi High-Tech Science DSC-7000, Tokyo, Japan) under a nitrogen atmosphere. Each sample of 4–6 mg was heated at a rate of 10 °C min^−1^ from 30 to 200 °C and held at 200 °C for 5 min in the first heating process to remove any thermal history. The sample was cooled to −50 °C at a rate of 10 °C min^−1^ and kept at −50 °C for 5 min. In the second heating process, the sample was heated from −50 to 200 °C at a rate of 10 °C min^−1^. The glass transition temperature, cold crystallization temperature, melting temperature, enthalpy of cold crystallization, and enthalpy of melting were recorded for each sample.

DSC (Hitachi High-Tech Science DSC-7000, Tokyo, Japan) was also employed to evaluate the nonisothermal crystallization behavior of various polymer blends. The prepared samples were kept in aluminum pans under nitrogen atmosphere. The samples were first heated from 30 to 200 °C at 10 °C min^−1^ and then maintained at 200 °C for 5 min to remove any thermal history. Next, the samples were cooled to 50 °C at various rates of 2.5, 5, 7.5, and 10 °C min^−1^. Finally, heat flow traces were recorded for analysis.

#### 2.3.4. Mechanical Testing

The tensile and impact strength for various polymer blends were measured in this study. A universal material testing machine (Cometech QC-508M2F equipment, Taichung, Taiwan) was used. The tensile tests were conducted at a tensile rate of 50 mm min^−1^ using dumbbell specimens (ASTM D638 Type IV, length: 33 mm, width: 6 mm, thickness: 3 mm, the gauge length being 50 mm). The impact tests (ASTM D412; the degree between pendulum and sample was set at 150°) were performed using a Ceast pendulum impact tester (Model Resil 50B) and a Ceast notch cutting machine. The Izod method was employed. A 2.75 J hammer was sufficient to break all specimens tested for impact strength. At least five repeated tests were conducted for tensile and impact strength measurements. The averaged values and standard deviations for these mechanical properties were recorded.

#### 2.3.5. Surface Morphology

A scanning electron microscope (SEM, TESCAN VEGA 3 SBH, Brno, Czech Republic) was used to observe the surface morphology and the dispersion of nHA in the PLA/PBS/PBAT/nHA blends. The SEM was operated at an acceleration voltage of 20 kV and a magnification of 3000×. All samples were dried under vacuum and sputter-coated with gold before SEM experiments. The chemical composition of the blends was studied using an energy-dispersive X-ray spectroscope (EDX) attached to the SEM (Nova NanoSEM 230, currently supported by Thermo Fisher, Waltham, MA, USA).

#### 2.3.6. TEM Measurements

A transmission electron microscope (TEM, Hitachi H-7650, Tokyo, Japan) was used to investigate the distribution of nHA in the PLA/PBS/PBAT/nHA blends. Ultrathin sections of sample were prepared using a cryomicrotome with a diamond knife in a dry N_2_ atmosphere at −100 °C. The thin film pieces were placed on a copper grid and the TEM analyses were conducted at a low voltage of 70 kV to prevent electron beam damage.

#### 2.3.7. Thermogravimetric Analysis (TGA)

The thermogravimetric analyses for the polymer blends were performed using a thermogravimetric analyzer (TGA, Pyris 6, Perkin Elmer, New Castle, DE, USA) to investigate the thermal stability of the blended samples. Approximately 8 mg of each polymer blend sample was heated from 40 to 600 °C under a nitrogen atmosphere at a heating rate of 10 °C min^−1^. TGA thermograms were recorded during the heating process and differential thermogravimetric analyses (DTGAs) were also performed.

#### 2.3.8. Water Absorption Measurements

The water absorption for various polymer blends was investigated in this study. The specimens with 20 mm × 10 mm × 2 mm were dried in a vacuum oven at 50 °C for 48 h. The specimens were weighed after the drying process using a balance (Precisa ES 225 SM-DR, Dietikon, Switzerland, readability 0.01 mg). The specimens were then completely immersed in distilled water at 25 °C for 24 h and longer, up to 8 days. After immersing the specimens in distilled water for t days (t = 1, 2, 5, and 8), they were taken out and wiped with filter paper before weighing for water absorption. The water absorption percentages for various samples were determined from weight gain after the water immersion process.

## 3. Results and Discussion

### 3.1. Tensile Strength Test Results for PLA/PBS/PBAT Blends

The mechanical strength of polymer blends without the addition of nHA was first tested to screen for acceptable formulations. The tensile strength test results for these polymer blends without nHA are presented in Table 3. It is observed that specimen PLA60 had a maximum tensile stress (tensile strength) of 37.6 ± 1.8 MPa, while specimens A0 and B0 both had maximum tensile stress greater than 45 MPa. The tensile strength of human cortical and cancellous bone, although age-dependent, is 50–150 and 10–100 MPa, respectively [10,45,46]. According to the literature [42], tensile strength higher than 40 MPa meets the need for bone tissue engineering material. It is observed from Table 3 that specimens A0 and B0 almost reached the lower limit of the tensile strength of human cortical bone. The elongations at maximum tensile stress were about 8%. The tensile modulus results for specimens A0 and B0 were also better than that of human cancellous bone [8]. Therefore, PLA-based polymer blends containing 70 or 80 wt% PLA in this study were considered acceptable biomaterials for bone grafts. Specimens in series A and B listed in Table 2 were used for further physical and mechanical property analyses.

### 3.2. FTIR Measurement Results for PLA/PBS/PBAT/nHA Blends

**Figure 1:**
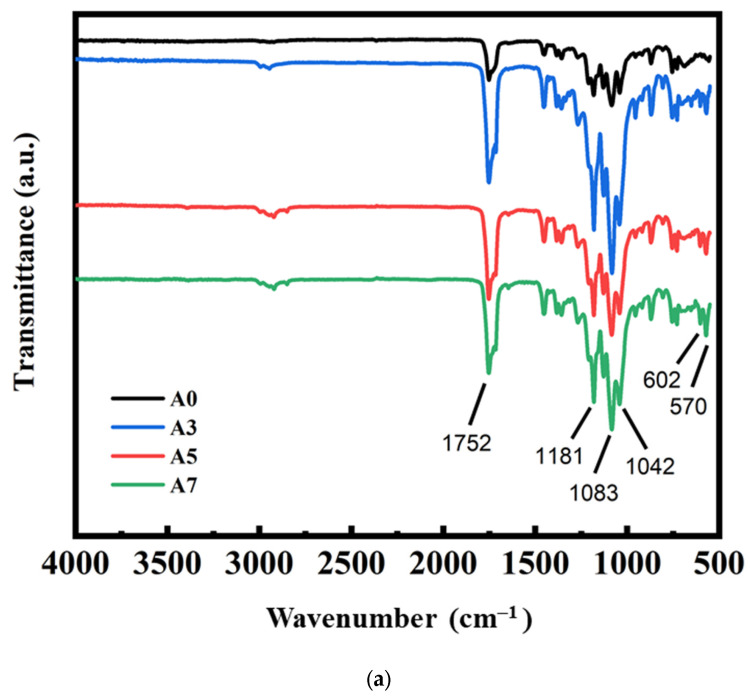

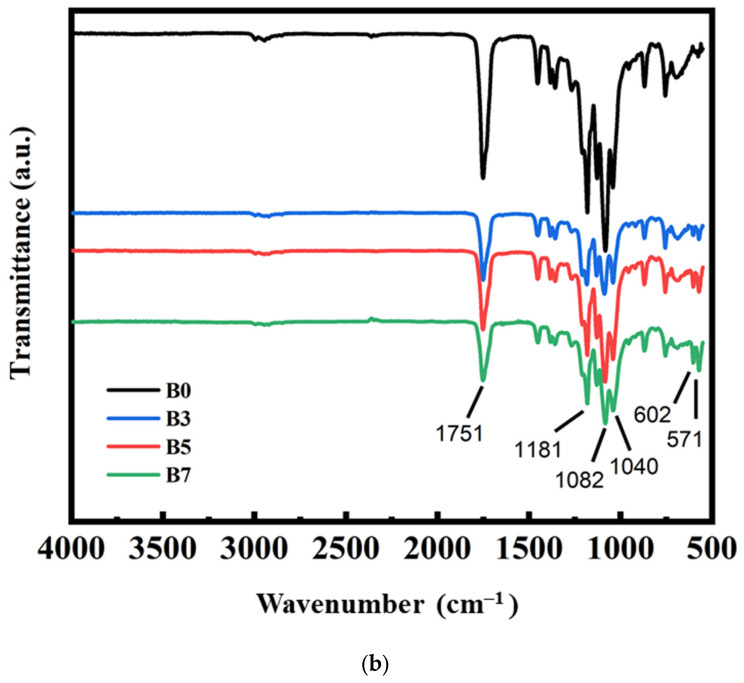
FTIR measurement results for polymer blends: (**a**) sample series A and (**b**) sample series B, with various amounts of nHA added.

The chemical structures for various PLA/PBS/PBAT/nHA blends were characterized using FTIR spectroscopy. The results for all specimens are presented in Figure 1. The band around the wavenumber of 3000 cm^−1^ represented the symmetric and asymmetric stretching vibration of CH_3_ in PLA. The peak at 1750 cm^−1^ showed the stretching of the C=O group of PLA as well as PBS and PBAT. The bands at 1181 cm^−1^ and 1082 cm^−1^ belonged to the peaks of C-O-C. The peak at 1040 cm^−1^ is attributed to C-CH_3_ [42,47,48,49,50]. The characteristic peaks at 602 cm^−1^ and 570 cm^−1^ represented the characteristic peaks of PO_3_^−4^ in nHA [42,48,50].

It was observed that no nHA peaks were present in specimens A0 and B0, to which no nHA particles were added during the preparation of the polymer blends. The characteristic peaks of nHA in the FTIR spectra for other specimens in Figure 1a,b clearly indicated the existence of nHA in the blends.

### 3.3. XRD Measurement Results for PLA/PBS/PBAT/nHA Blends

**Figure 2:**
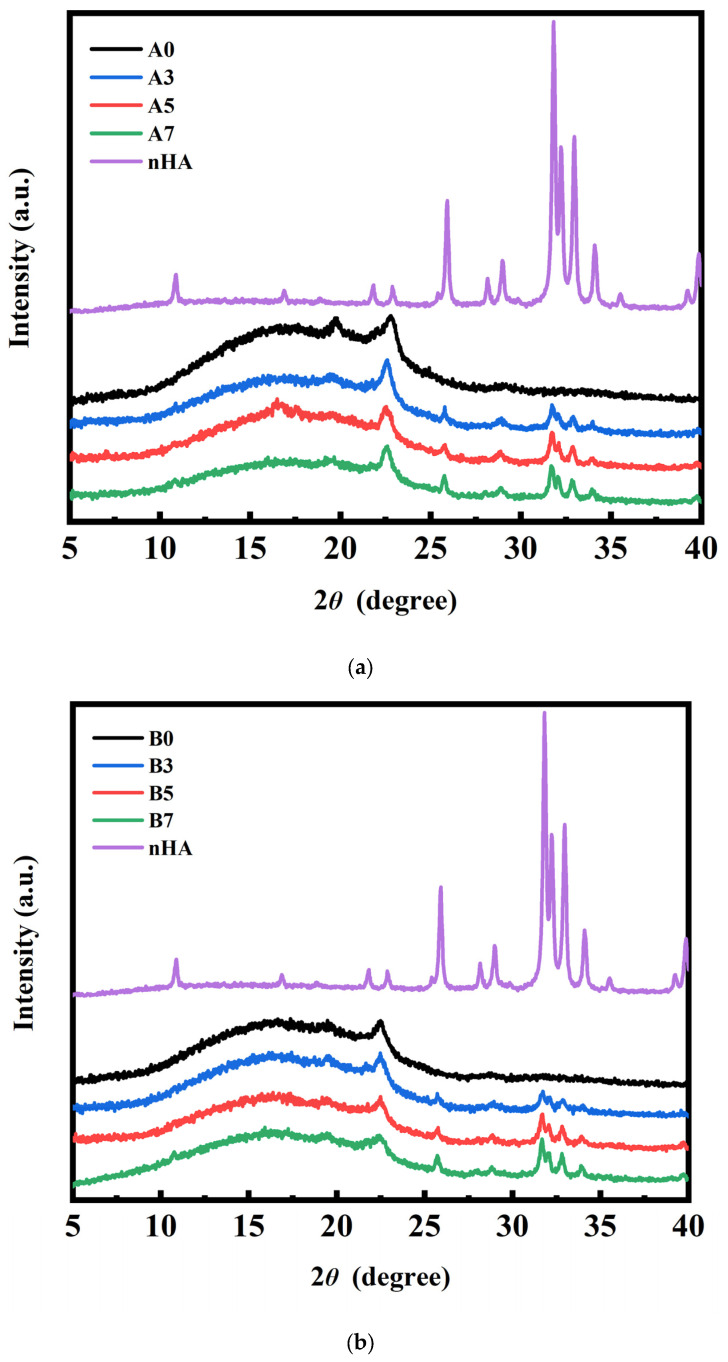
XRD measurement results for polymer blends: (**a**) sample series A and (**b**) sample series B, with various amounts of nHA added.

XRD analysis was performed to further confirm the presence of nHA in the PLA/PBS/PBAT/nHA polymer blends and to identify the crystalline species in the polymer blends. The results are presented in Figure 2a,b for sample series A and B with various amounts of nHA added. The diffraction peaks between 2 θ at 15° and 20° were owing to the characteristic pattern of PLA. The diffraction peaks of PBS were observed at 22.5°. The diffraction peaks at 25.9°, 28°, and 32–35° contributed to the characteristic peaks of nHA [42,49,50]. There were no nHA peaks for specimens A0 and B0 without added nHA in the PLA/PBS/PBAT blends. It is also shown that the characteristic peaks of nHA were observed for specimens A3 to A7 and B3 to B7.

### 3.4. DSC and Crystallization Analysis Results for PLA/PBS/PBAT/nHA Blends

**Figure 3:**
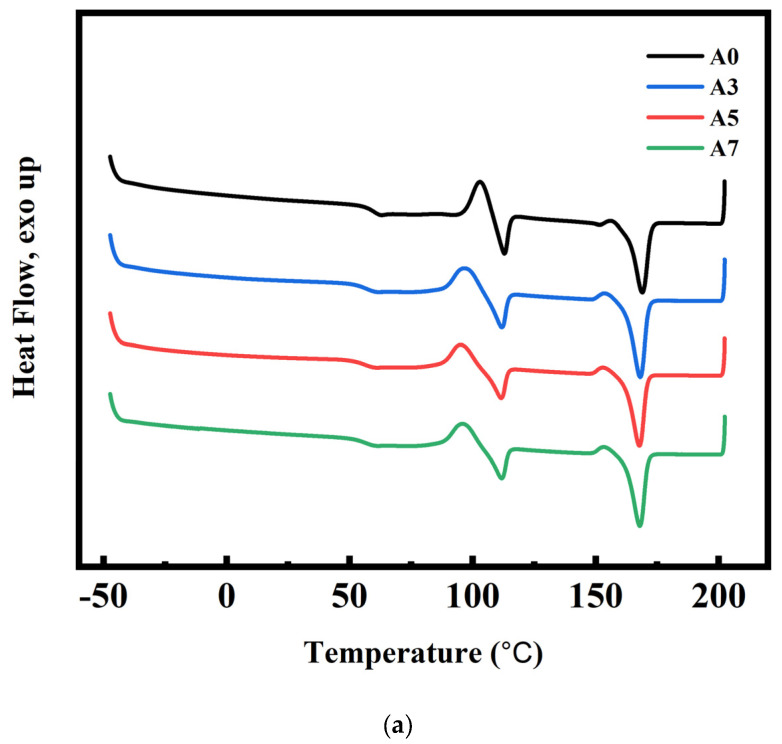

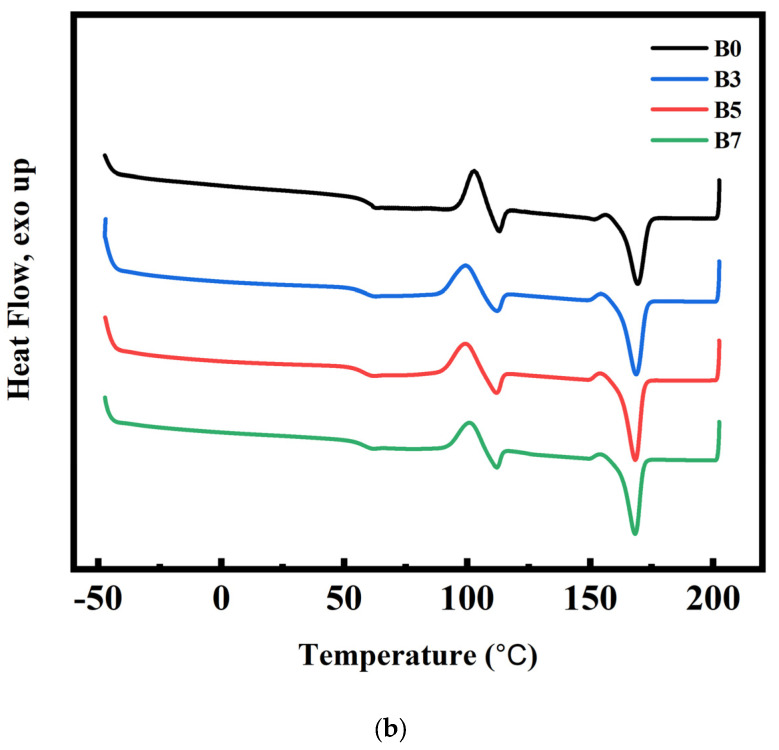
DSC curves (second heating) for polymer blends: (**a**) sample series A and (**b**) sample series B, with various amounts of nHA added.

DSC analyses were used to measure the reliable thermal history and crystallization of semi-crystalline polymers. The second heating curves of the DSC measurements for sample series A and B with various amounts of nHA added are shown in Figure 3a,b. The glass transition temperature of PLA (T_g,PLA_), cold crystallization temperature (T_cc_), melting temperature of PLA (T_m,PLA_), enthalpy changes of cold crystallization (ΔH_cc_), and enthalpy changes of melting of blends (ΔH_m_), as listed in Table 4, were retrieved from the DSC thermogram. Addition of nHA affected the crystallization behavior of the PLA/PBS/PBAT blends and, therefore, their physical properties [42,51]. The relative crystallinity X_c_ of blends with various formulations was calculated by:

(1) $$X_{c}=\frac{∆H_{m}-∆H_{cc}}{\omega_{PLA}{∆H}_{o,PLA}+\omega_{PBS}{∆H}_{0,\;PBS}+\omega_{PBAT}{∆H}_{0,PBAT}}\times 100\%$$

where $\omega_{PLA},\;\omega_{PBS}$, and $\omega_{PBAT}$ represented the weight fractions of PLA, PBS, and PBAT in the polymer blends, respectively. Δ*H*_0_*_,__PLA_*, Δ*H*_0_*_,__PBS_*, and Δ*H*_0_*_,__PBAT_* denoted the enthalpy of fusion values for 100% crystalline polymers. The theoretical values Δ*H*_0_ of PLA, PBS, and PBAT are 93.1, 200, and 114 J g^−1^, respectively [42,51,52,53,54].

As discussed in the literature, the thermodynamic compatibility between PLA and PBS or PLA and PBAT was poor [26,51,55,56,57]. Nanoparticles were used as a nucleating agent in the crystallization process to increase crystal growth [55,56,57,58]. As shown in Table 4, the relative crystallinity X_c_ in the PLA/PBS/PBAT blend (specimen A0) was 20.1%. The relative crystallinity X_c_ in polymer blends with the addition of various amounts of nHA (specimens A3, A5, and A7) increased from 24.8 to 27.0%. Increased crystallinity of polymer blends would lead to better mechanical properties. It is observed from Table 4 that sample series B (specimens B0, B3, B5, and B7) also had a similar slight increase in relative crystallinity X_c_ as the added amount of nHA increased.

### 3.5. Nonisothermal Crystallization Behavior of PLA/PBS/PBAT/nHA Blends

**Figure 4:**
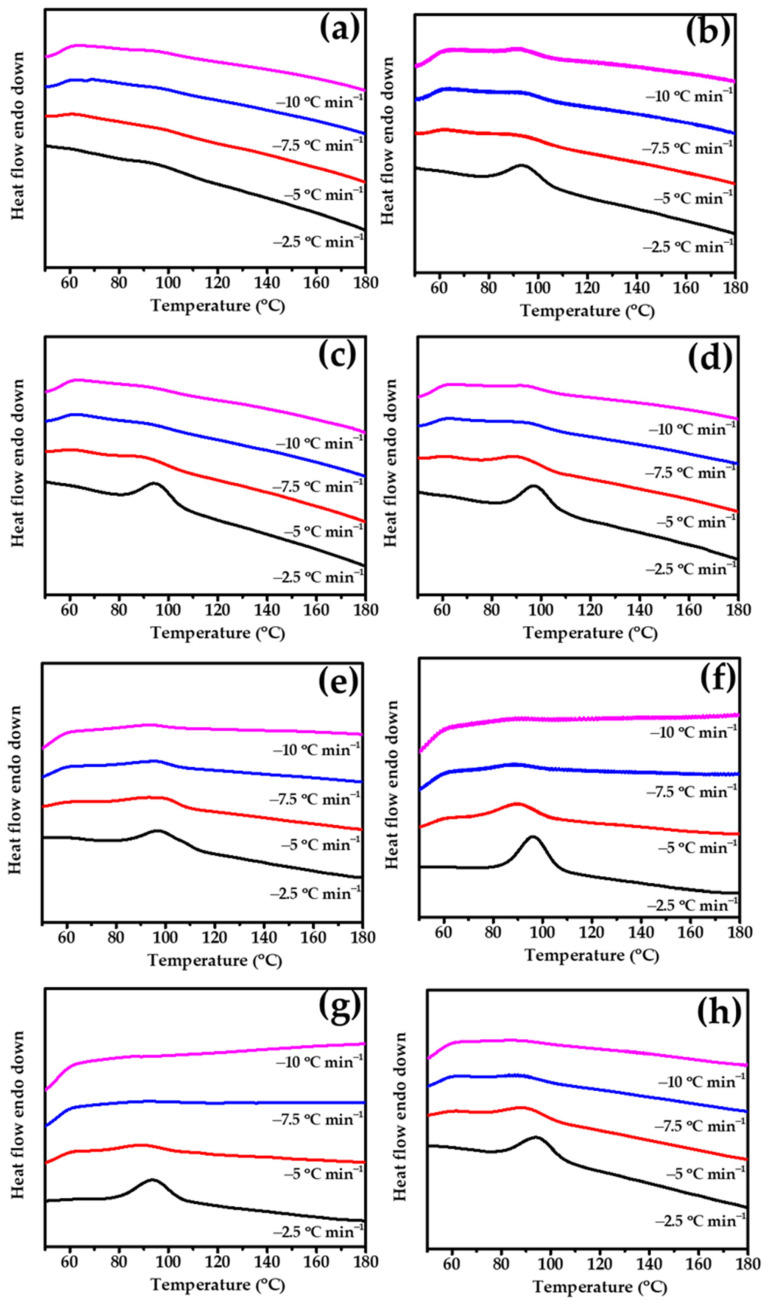
DSC curves of prepared specimens in the cooling process at 2.5, 5, 7.5, and 10 °C min^−1^ (**a**) A0, (**b**) A3, (**c**) A5, (**d**) A7, (**e**) B0, (**f**) B3, (**g**) B5, and (**h**) B7.

Heterogeneous nucleation effects in nonisothermal crystallized polymer nanocomposites characterized by DSC have been discussed in the literature [59,60]. Figure 4 shows the heat flow curves of the prepared specimens in this study during cooling from 200 °C to 50 °C at rates of 2.5, 5, 7.5, and 10 °C min^−1^.

The cooling traces showed that no obvious peak was observed in specimen A0, and its melt crystallization ability was poor. When the nHA content in the specimen increased from 0 to 3 wt%, the appearance of crystallization behavior was observed during the cooling process at 2.5 °C min^−1^, which exhibited an increase in the crystallization enthalpy value ΔH_hc_ from 0 to 7.8 J g^−1^. This result can be attributed to the introduced nHA, which may play a role as a nucleating agent and lead to heterogeneous nucleation behavior. As the nHA content further increased, the ΔH_hc_ values increased slightly. Compared with specimen A0, specimen B0 showed a relatively high melt crystallization ability, exhibiting a ΔH_hc_ value of 10.1 J g^−1^ at a cooling rate of 2.5 °C min^−1^ in the absence of nHA. However, similar ∆H_hc_ values were observed in B3, B5, and B7 specimens, indicating that no significant crystallinity change was obtained upon the addition of nHA.

All nonisothermal DSC-measured data are listed in Table 5. The different tendencies of the sample series A and B may result in the basic crystallization ability of PLA in different compositions. The PLA content in specimen A0 was lower, resulting in limited melt crystallization during the cooling process. When nHA was added, a nucleation effect occurred, making crystal formation less difficult. Therefore, as the nHA content increased, the ΔH_hc_ increased significantly. On the other hand, specimen B0 had a relatively higher PLA content and had advantages in crystallization. The addition of nHA in sample series B may not have produced significant improvements in crystallization compared to neat specimen B0 without nHA.

### 3.6. Tensile and Impact Strength Test Results for PLA/PBS/PBAT/nHA Blends

**Figure 5:**
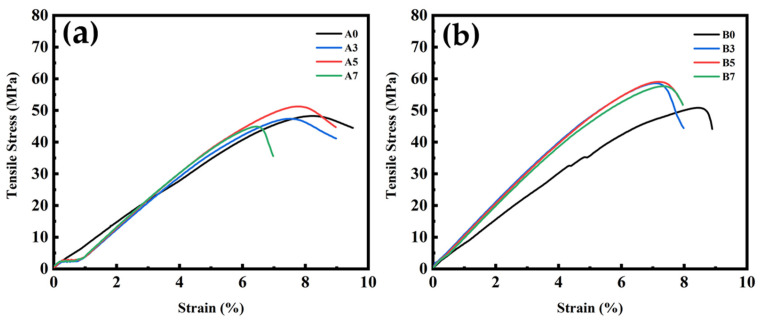
Typical tensile stress measurement results for polymer blends: (**a**) sample series A and (**b**) sample series B, with various amounts of nHA added.

Tensile and impact strength tests were conducted in this study to examine the mechanical properties of various polymer blends. Typical tensile stress measurement results of sample series A and B with various amounts of nHA added are graphically shown in Figure 5. The maximum tensile stress (tensile strength), elongation at maximum tensile stress, tensile modulus, and impact strength data obtained from repeated tests in this study are listed in Table 6. It was observed that sample series B containing 80 wt% PLA exhibited higher tensile strength, up to 57.9 ± 1.0 MPa, while the tensile strength of sample series A with 70 wt% PLA was about 47 to 50 MPa. The elongation of sample series A and B both ranged from 7 to 8%, better than neat PLA (6%, as shown in Table 1) or human bone (1–3% for cortical bone and 3–7% for cancellous bone) [8]. The tensile modulus for sample series A with various nHA contents ranged from 642 to 905 MPa, while that of sample series B could reach 1.0 GPa. Tensile strength and tensile modulus increased with increasing nHA addition, reaching the highest values for polymer blends containing 5 wt% nHA (specimens A5 and B5). Polymer blends with a higher 7 wt% nHA (specimens A7 and B7) showed a slight decrease in tensile strength, suggesting that there might be an optimal nHA addition. Impact strength measurements indicated the toughness of the polymer blend. The impact strength of neat PLA was about 2.5 kJ m^−2^ [21]. All polymer blends yielded better toughness by blending PLA with ductile PBS and PBAT. It can be seen from Table 6 that the additional amount of nHA in this study had no significant effect on the impact strength results for a specific sample series. Examination of these comparisons indicated that specimens B3 or B5 could be the formulations with acceptable mechanical strength for composite bone graft materials. It was deduced from the mechanical property measurements that the added nHA nanoparticles acted as nucleating agents during the crystallization process of the polymer blends, thereby increasing the crystallinity and tensile strength.

### 3.7. SEM Analysis Results for PLA/PBS/PBAT/nHA Blends

**Figure 6:**
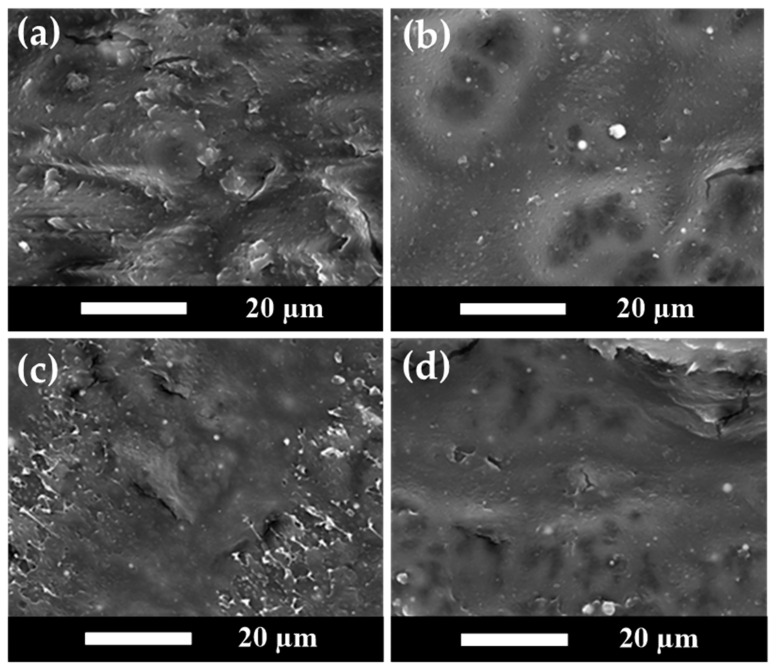
Comparison of the SEM images for polymer blend sample series A with various amounts of nHA added in (**a**) Specimen A0, (**b**) Specimen A3, (**c**) Specimen A5, and (**d**) Specimen A7.

**Figure 7:**
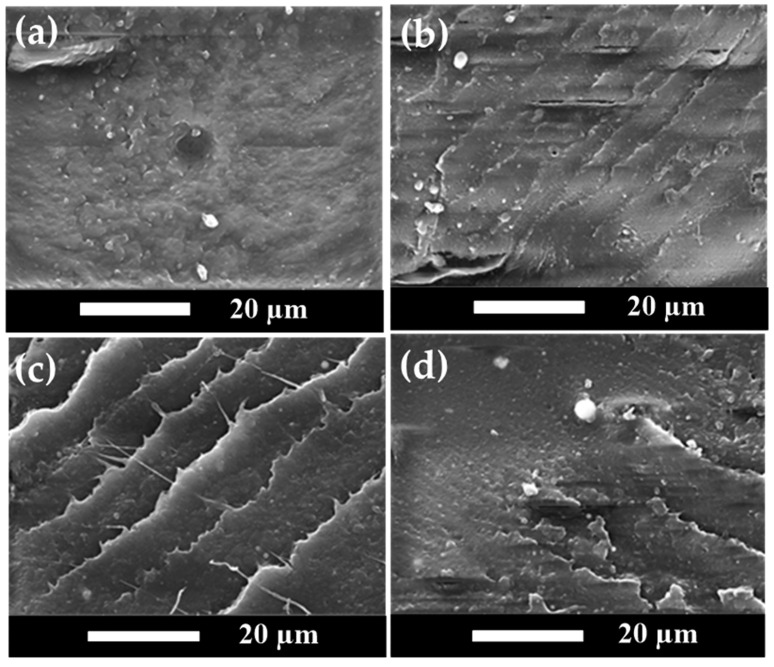
Comparison of the SEM images for polymer blend sample series B with various amounts of nHA added in (**a**) Specimen B0, (**b**) Specimen B3, (**c**) Specimen B5, and (**d**) Specimen B7.

SEM analyses of fractured surfaces have been demonstrated in the literature discussing polymer composite morphology and additive distribution in the polymer matrix [61,62,63]. The transition from ductile to brittle in polymer composites and the morphology–mechanical properties relationship can be examined using SEM analyses. SEM images of the impact-fractured surfaces for sample series A and B of polymer blends in this study are shown in Figure 6 and Figure 7, respectively.

Figure 6a shows the SEM image of specimen A0. Figure 6b–d show the SEM images of specimens A3, A5, and A7, respectively. Figure 6a indicates that specimen A0 without nHA presented a relatively rough and tough fractured surface. For specimen A3 with 3 wt% nHA added, well-dispersed nHA particles can be observed as small white dots in Figure 6b. With the higher amount of added nHA, a brittle fractured morphology with cracks appeared, as shown in Figure 6c,d. A transition in fractured surface morphology from relatively rough and ductile to hard and brittle was observed. These SEM results are also supported by the TEM results discussed in the next section, showing that the nanoparticles were uniformly distributed. The effect of nHA addition on tensile and impact strength was consistent with the SEM results, where the polymer blends became harder and brittle with increasing nHA addition. As the amount of nHA added increased, the mechanical tensile strength showed an increasing trend.

**Figure 8:**
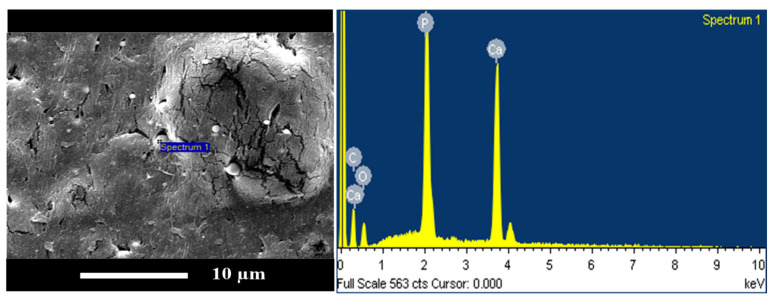
Typical results of the EDX spectra of a polymer blend (specimen B7, with the addition of 7 wt% nHA).

Figure 7 shows similar SEM images for sample series B, containing 80 wt% PLA. An impact-fractured surface morphology consistent with Figure 6 was observed. No nHA particles were detected in Figure 7a, which presented a relatively rough surface. When nHA particles were added to the polymer blends, they produced sharp fractured surface morphologies, as shown in Figure 7b–d. The nHA particles were well dispersed in the polymer matrix and became aggregated at higher nHA contents. As the amount of nHA added increased, the fracture morphology once again exhibited hard and brittle characteristics. To confirm the chemical compositions of nanoparticles shown in the SEM images, the EDX spectrum for a typical polymer blend of specimen B7 is shown in Figure 8. The characteristic peaks of Ca and P elements shown in Figure 8 indicated the nHA particles in specimens.

### 3.8. TEM Measurement Results for PLA/PBS/PBAT/nHA Blends

**Figure 9:**
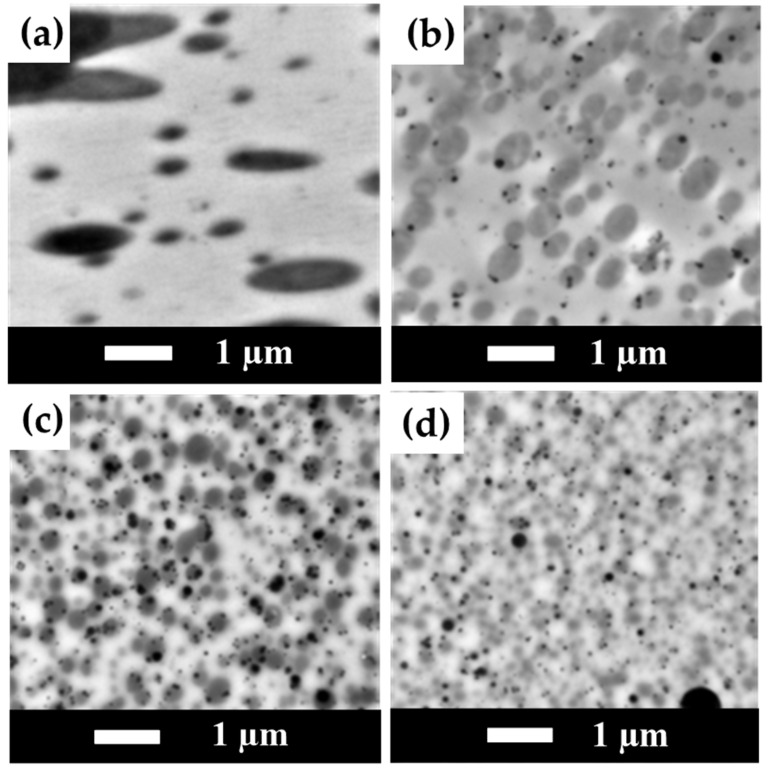
TEM images for polymer blends of sample series A with various amounts of nHA added (magnification 20,000×). (**a**) Specimen A0, (**b**) Specimen A3, (**c**) Specimen A5, and (**d**) Specimen A7.

**Figure 10:**
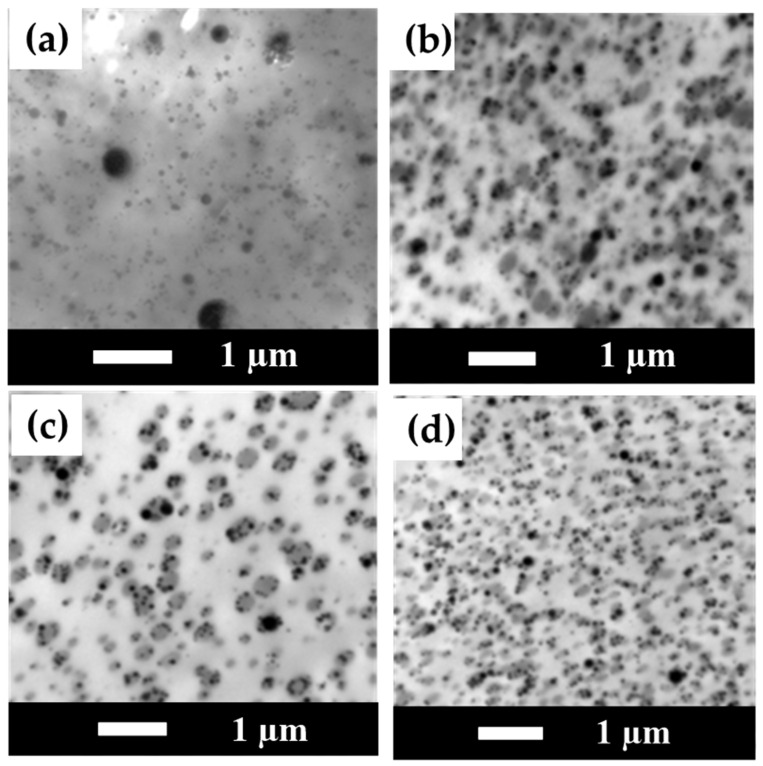
TEM images for polymer blends of sample series B with various amounts of nHA added (magnification 20,000×). (**a**) Specimen B0, (**b**) Specimen B3, (**c**) Specimen B5, and (**d**) Specimen B7.

TEM images of sample series A are shown in Figure 9 at 20,000× magnification. No nHA particle images were detected in specimen A0. In addition, phase separation morphology in the blend can be observed in Figure 9a, with a continuous region (or lighter region) as the major component PLA matrix and a darker region as the minor PBS/PBAT dispersed phase. With the addition of 3 wt% nHA, the TEM image shown in Figure 9b exhibits the presence of small black dots of nHA, with a particle size in the range of 70–100 nm. Furthermore, the PBS/PBAT dispersed phase became smaller in size with the addition of nHA particles, indicating that the addition of nHA particles helped to disperse polymer phases during the twin-screw mixing process. With a further increased amount of nHA addition from 5 to 7 wt%, the degree of uniform dispersion of the polymer phases also increased, and PBS/PBAT phases became even smaller, as seen in the images shown in Figure 9c,d. However, it is noted that with a higher amount of nHA addition, nHA particles became larger due to aggregation during mixing. For 7 wt% of nHA addition, a near-micrometer size of nHA particles was observed, as seen in the image shown in Figure 9d. Similar results for TEM images of sample series B are presented in Figure 10. For specimen B0, no nHA particle images were detected, as seen in Figure 10a. When the addition of nHA increased from 3 to 7 wt% as shown in Figure 10b–d, an increased number of fine black dots of nHA particles was displayed. TEM images also showed nHA-induced morphological refinement, indicating that nHA helped disperse polymer domains.

### 3.9. TGA for PLA/PBS/PBAT/nHA Blends

**Figure 11:**
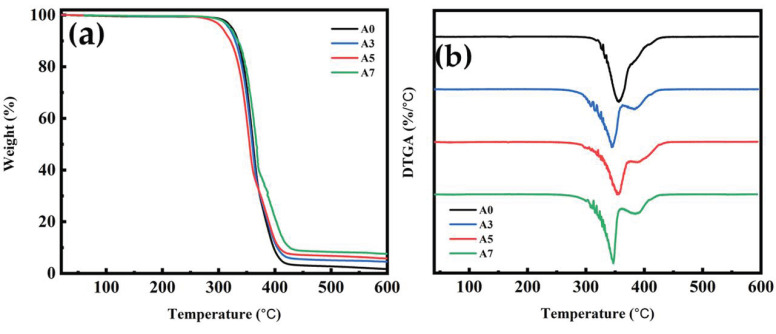
Thermogravimetric analysis results of (**a**) TG and (**b**) DTG thermograms for sample series A, with various amounts of nHA added.

**Figure 12:**
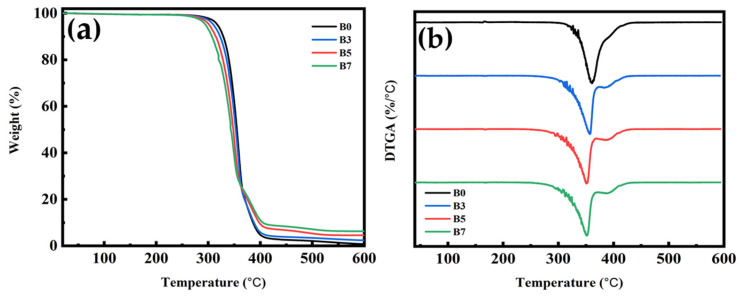
Thermogravimetric analysis results of (**a**) TG and (**b**) DTG thermograms for sample series B, with various amounts of nHA added.

This study examined the thermal stability of polymer blends with different formulations using thermogravimetric analysis (TGA). The results for sample series A and B are shown with TGA and DTGA thermograms in Figure 11 and Figure 12, respectively. Table 7 lists the decomposition temperatures for 5% and 50% weight loss (T_5%_ and T_50%_), the temperature of maximum decomposition rate (T_max_), and residual weight percent at 600 °C (W_R_) for all specimens measured in this study. For sample series A, the TGA thermograms in Figure 11a show that the main decomposition temperature of each specimen (A0 to A7) was between 300 and 400 °C. The DTGA thermograms shown in Figure 11b depict the two-step decomposition of each specimen. The first peak indicated the maximum decomposition rate of PLA [64], and the second peak was owing to the maximum decomposition rate of PBS/PBAT. As shown in Figure 11 and Table 7, adding nHA did not seem to significantly affect the T_max_ of the polymer blends. The residual weight percent in the temperature range of 500 to 600 °C increased reasonably well with increasing thermally stable inorganic nHA content, as shown in Figure 11a and Table 7. The similar TGA and DTGA thermograms of sample series B are shown in Figure 12, with data also listed in Table 7. The main decomposition temperature range was also between 300 and 400 °C, as shown in Figure 12a. The increase in residual weight percent for four specimens of sample series B was again consistent with the increasing amount of nHA added. From the TGA results, polymer blends in this study all had good thermal stability for bone graft material applications.

### 3.10. Water Absorption Measurement Results for PLA/PBS/PBAT/nHA Blends

**Figure 13:**
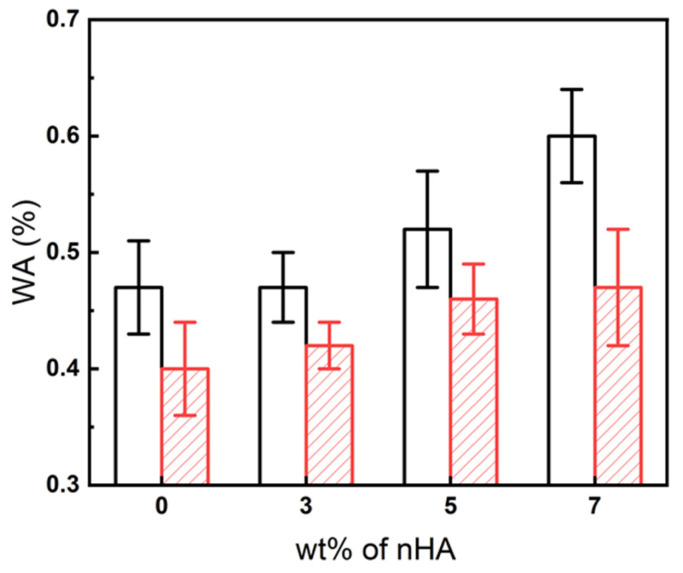
Comparison of water absorption percentage gain (WA) at 24 h for all specimens with various amounts of nHA added. (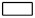: sample series A; 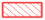: sample series B).

**Figure 14:**
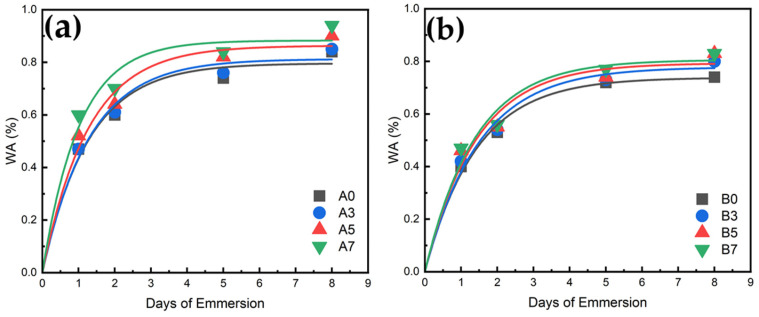
Water absorption profiles for (**a**) sample series A and (**b**) sample series B.

The water absorption percentage gain (WA) was calculated by:

(2) $$WA\left(\%\right)=\frac{W_{t}-W_{d}}{W_{d}}\times 100\%$$

where *W_d_* was the weight of the sample after drying (dried at 50 °C for 2 days before the water absorption test) and *W_t_* was the weight of the sample measured after immersion in distilled water for t days (t = 1, 2, 5, and 8). The water absorption results for all specimens after immersion in distilled water for 24 h are shown in Figure 13. For sample series A, due to the hydrophilicity of nHA, the water absorption tended to increase with the increase in nHA addition. Sample series B also exhibited a similar water absorption trend. Compared to sample series A, sample series B displayed lower water absorption due to its higher PLA content. For the 24 h test, the water absorption of specimens B5 and B7 was about 15% better than that of specimen B0. The water absorption results for a longer time are depicted in Figure 14 for all specimens. It is demonstrated that after five days of water immersion, the degree of water absorption of each specimen increased by about 40% to 50% compared to the degree of water absorption on the first day. After absorbing water for eight days, the degree of water absorption increased by about 70 to 80% compared with one-day water absorption results. These increased water absorption percentages were consistent with those shown in the literature for composites involving PLA [65,66]. The results of water absorption indicated that the addition of hydrophilic nHA enhanced the water absorption rate, which is beneficial to the application of polymer blends in bone tissue engineering.

## 4. Conclusions

The physical and mechanical properties of novel bio-based polymer blends of PLA/PBS/PBAT/nHA were investigated in this study. The aim was to screen promising formulations of polymer blends with biocompatible and osteoconductive nHA to provide options for bone graft materials. Various analytical results for physical and mechanical property tests were reported. The conclusions are as follows: (1) Polymer blend formulations containing 70 or 80 wt% PLA content with the addition of 3 to 5 wt% nHA exhibited tensile strengths ranging from 47 to 57 MPa, suitable for bone graft applications. The elongation and impact strength of these formulations were also satisfactory. (2) The thermal degradation temperatures of various PLA/PBS/PBAT/nHA blends ranged from 300 to 400 °C, suggesting that the polymer blends had satisfactory thermal stability. (3) The addition of nHA in the polymer blends increased water absorption due to its hydrophilicity. Addition of 5 wt% nHA resulted in about a 15% increase in water absorption in the 24 h water immersion test compared to polymer blends without nHA.

## Figures and Tables

**Table 1:** Physical and mechanical properties of polymers used in this study ^a^.

Physical Properties	PLA	PBS	PBAT
Density (g/cm^3^)	1.24	1.26	1.25–1.27
Melt flow index (g/10 min, at 190 °C, 2.16 kg)	7	5	2.7–4.9
Melting temperature, T_m_ (°C)	155–170	115	110–120
Tensile yield strength (MPa)	60	39	36
Tensile modulus (GPa)	3.5	0.58	–
Tensile elongation (%)	6.0	350	560

^a^: according to the suppliers.

**Table 2:** Formulations of PLA/PBS/PBAT/nHA blends in this study.

Specimen Code	PLA (wt%)	PBS (wt%)	PBAT (wt%)	nHA (wt%)
Sample with 60 wt% PLA				
PLA60	60	25	15	0
Sample series A				
A0	70	20	10	0
A3	70	20	10	3
A5	70	20	10	5
A7	70	20	10	7
Sample series B				
B0	80	15	5	0
B3	80	15	5	3
B5	80	15	5	5
B7	80	15	5	7

**Table 3:** Tensile test results of PLA/PBS/PBAT blends.

Sample of PLA/PBS/PBAT Blends	Maximum Tensile Stress (MPa)	Elongation at Maximum Tensile Stress (%)	Tensile Modulus (MPa)
PLA60	37.6 ± 1.8	7.0 ± 0.5	584.8 ± 71.9
A0	47.1 ± 2.9	7.9 ± 0.4	642.0 ± 109.1
B0	49.6 ± 1.6	8.0 ± 0.6	686.1 ± 91.3

**Table 4:** DSC test results and the calculated degree of crystallinity for various polymer blends.

Specimen Code	T_g,PLA_ (°C)	T_cc_ (°C)	T_m,PLA_ (°C)	ΔH_cc_ (J g^−1^)	ΔH_m_ (J g^−1^)	X_c_ (%)
A0	62	103	169	12.0	36.3	20.1
A3	63	100	169	13.0	41.9	24.8
A5	63	99	168	14.9	45.2	26.0
A7	61	99	168	13.5	45.0	27.0
B0	62	103	169	15.7	37.9	20.1
B3	63	101	169	18.6	41.3	20.6
B5	63	102	169	15.6	40.2	22.3
B7	63	102	169	14.8	38.5	21.5

**Table 5:** Crystallization enthalpy values of sample series A and B under various cooling rates.

Specimen Code	Cooling Rate (°C min^−1^)			
	2.5	5	7.5	10
	∆H_hc_ (J g^−1^)			
A0	0	0	0	0
A3	7.80	1.08	0.63	0.37
A5	9.88	1.70	0.45	0.41
A7	10.90	2.72	0.91	0.50
B0	10.12	3.46	0.74	0.55
B3	11.96	3.31	0.94	0.46
B5	10.09	2.56	0.84	0.36
B7	10.91	2.71	0.94	0.48

**Table 6:** Tensile and impact test results of polymer blends with various compositions of nHA.

Specimen Code	Maximum Tensile Stress (MPa)	Elongation at Maximum Tensile Stress (%)	Tensile Modulus (MPa)	Impact Strength (kJ m^−2^)
A0	47.1 ± 2.9	7.9 ± 0.4	642.0 ± 109.1	4.83 ± 0.19
A3	47.5 ± 0.7	7.5 ± 0.3	887.8 ± 57.2	4.32 ± 0.17
A5	50.2 ± 1.0	7.4 ± 0.4	905.5 ± 64.1	4.64 ± 0.21
A7	47.4 ± 0.3	6.7 ± 0.3	707.5 ± 15.0	4.20 ± 0.07
B0	49.6 ± 1.6	8.0 ± 0.6	686.1 ± 91.3	3.36 ± 0.20
B3	57.8 ± 0.7	7.1 ± 0.2	1039.4 ± 37.5	3.21 ± 0.16
B5	57.9 ± 1.0	7.3 ± 0.2	1062.2 ± 97.8	3.42 ± 0.17
B7	56.7 ± 1.0	7.3 ± 0.1	962.6 ± 17.4	3.19 ± 0.25

**Table 7:** Thermogravimetric measurement results for PLA/PBS/PBAT/nHA blends.

Specimen Code	T_5%_ (°C)	T_50%_ (°C)	T_max_ (°C)	W_R_ (%) at 600 °C
A0	325	361	361	1.6
A3	318	360	360	4.5
A5	306	354	355	5.7
A7	319	367	366	7.6
B0	317	355	371	0.7
B3	310	353	369	2.3
B5	302	348	362	4.5
B7	295	343	352	6.3

## Data Availability

Data are contained within the article.
